# Structured Water Dance Intervention (SWAN) for adults with profound intellectual and multiple disabilities: study protocol

**DOI:** 10.1016/j.heliyon.2020.e04242

**Published:** 2020-07-25

**Authors:** Lars-Olov Lundqvist, Marie Matérne, Anette Granberg, André Frank, Patrik Arvidsson, Anna Duberg

**Affiliations:** a University Health Care Research Center, Faculty of Medicine and Health, Örebro University, SE 70182, Örebro, Sweden; b Region Gävleborg, Centre for Research & Development, Uppsala University/Region Gävleborg, 806 33, Gävle, Sweden

**Keywords:** Rehabilitation, Quality of life, Disability, Physical activity, Musculoskeletal system, Health psychology, Well-being, Profound intellectual multiple disabilities, Intervention, Randomized clinical trial, Health-related quality of life, Study protocol

## Abstract

**Background:**

People with profound intellectual and multiple disabilities (PIMD) have a combination of severe intellectual disability, extensive physical impairment, sensory impairments and medical health problems. There is, however, a lack of evidence-based physical and health-promoting interventions for people with PIMD.

**Objective:**

Structured Water Dance Intervention (SWAN) is a new method developed to fill this gap. This paper reports a protocol for an intervention study which aims to evaluate SWAN with regard to its effects on physiological, psychological and social health-related variables as well as its cost-effectiveness and potential for implementation in health care.

**Methods:**

The evaluation of SWAN is performed in a multi-center randomized crossover study. Data is collected through cortisol measurement, physiological assessments, proxy ratings, video observations and interviews.

**Conclusions:**

This is the first attempt to evaluate rigorously an innovative intervention for people with PIMD, a group that is rarely considered for health promotion interventions. This study will provide important information about the efficacy, cost-effectiveness and potential to implement SWAN in health care.

**What this paper adds**

This paper describes the SWAN evaluation study rationale and design, and provide information on the effects and efficacy of SWAN for people with PIMD as well as information on cost effectiveness and on obstacles and enabling factors for the implementation of SWAN in health care settings. The strengths of the study include randomization by minimization to manage participant heterogeneity, the use of multi-methods collecting both quantitative and qualitative data, the use of well-validated data collection methods, cost-effectiveness analyses and interviews with staff and managers to address opportunities and obstacles for the implementation of SWAN. It adds to the need to address people with PIMD and fill the research gap concerning customized interventions. It may be reasonable to assume that people with fewer resources will have similar needs and thus derive similar benefits from SWAN. Thus, if the objectives of the study are reached, it will provide a means to improve the health-related quality of life for people with PIMD, a group with substantial needs.

## 1 Introduction

People with profound intellectual and multiple disabilities (PIMD) have a combination of severe intellectual disability and physical impairment, as well as additional sensory impairments, epilepsy or major medical health problems [1]. People with PIMD also have orthopedic complications such as scoliosis and contractures, as well as pain problems that are difficult for staff and relatives to interpret. The causes of PIMD may be chromosomal abnormalities, congenital anomalies, asphyxia, and infection in the fetus or acquired brain injuries resulting from early onset disease or severe accidents. The prevalence of PIMD is low. The most recent figures from Sweden indicate that there were about 80 people per 100,000 inhabitants diagnosed with PIMD in 2010 [2]. In comparison, a study from 2016 carried out in the Netherlands estimated the prevalence there to be approximately 60 per 100,000 inhabitants [3].

Because of the extent of their symptoms, people with PIMD are dependent on personal assistance at all times [2]. Their limited communication capabilities make it difficult for them to convey feelings, thoughts and needs to others, and therefore they have great difficulties in maintaining their integrity and being involved in social interactions [1]. In many countries, people with PIMD are offered some form of organized daily activities. Research in the Netherlands showed that the activities offered can be short and passive in nature and that multiple sensory stimulation is the most popular therapy among activities such as massage, music or warm water pool activities [4]. There is no corresponding overview of the situation in Sweden, but it is reasonable to assume that it is similar.

Floor dance [5] is an activity involving movement to music for people with PIMD. In floor dance, the participant, who is lying on the floor, gets help from staff to move his or her body to music. Floor dance has its roots in dance therapy and is popular and widespread in habilitation centers in Sweden [5]. Floor dance has not been scientifically evaluated, but anecdotal statements from parents and staff testify to its positive effects. Another common activity available to people with PIMD in Sweden is hydrotherapy. Hydrotherapy involves treatments that use the physical properties of water, such as temperature and pressure, for therapeutic purposes [6]. Studies indicate that hydrotherapy can reduce heart rate, reduce pain and decrease anxiety [6], as well as decrease spasticity [7] and stress [8]. In addition, members of staff with experience of hydrotherapy for people with PIMD believe that it is a suitable form of activity for them but that specially designed programs are warranted [9].

### 1.1 Structured Water Dance Intervention

Based on the positive experiences of floor dance and hydrotherapy, physiotherapists at the Adult Rehabilitation Center in Örebro, Sweden, combined the advantages of the two methods in what came to be called Structured Water Dance Intervention (SWAN). Using specific body movements equivalent to dance steps, SWAN was designed to give the participants opportunities to enjoy movement and social interaction. By performing dance movements in a warm water pool, synergy effects were expected in terms of reduced spasticity, stress and pain and increased well-being. In 2017, a pilot study was conducted with five persons with PIMD [10]. The results of the pilot indicate that the participants’ spasticity decreased and their level of alertness was slightly increased during the intervention. All participants reached their pre-specified goals, in full or in part, after completing the intervention.

#### 1.1.1 Assessment of the project's scientific area of concern

SWAN is a method based on a health promotion and disease prevention approach in accordance with the national guidelines for disease prevention methods issued by the Swedish National Board of Health and Welfare [11]. People with PIMD cannot perform daily physical activity on their own. Because of their extensive disabilities, they are rarely considered for inclusion in various health-promoting initiatives, though they are at high risk of developing health problems. Therefore, it is important that interventions for them are customized, of high quality and evidence-based.

#### 1.1.2 Knowledge gaps the project intends to fill

Floor dance and hydrotherapy have been used in the rehabilitation of people with PIMD, but there are to our knowledge no published scientific studies on the effect of these methods on these individuals. To determine whether SWAN, which is inspired by these two methods, is an effective treatment, randomized controlled trials are needed. Using a multiple method approach including objective and subjective measures [12], the SWAN project aims to fill this gap. The knowledge generated in the study can be used by a variety of health service providers in order to implement SWAN for people with PIMD. It is also important to increase our knowledge of the implementation process and facilitate the implementation of evidence-based, cost-effective methods in clinical work. Therefore, the study covers the full intervention research process, from intervention design to implementation [13]. More specifically, the study objectives are:

⁃ to evaluate the effects of SWAN on health outcomes such as stress, spasticity, alertness, pain, well-being, quality of life and social interaction in people with PIMD.
⁃ to evaluate the cost-effectiveness of SWAN with respect to changes in healthcare consumption, health status and quality of life.
⁃ to investigate the opportunities and obstacles with the implementation of SWAN in the healthcare system.

## 2 Methods

### 2.1 Study design

**Figure 1:**
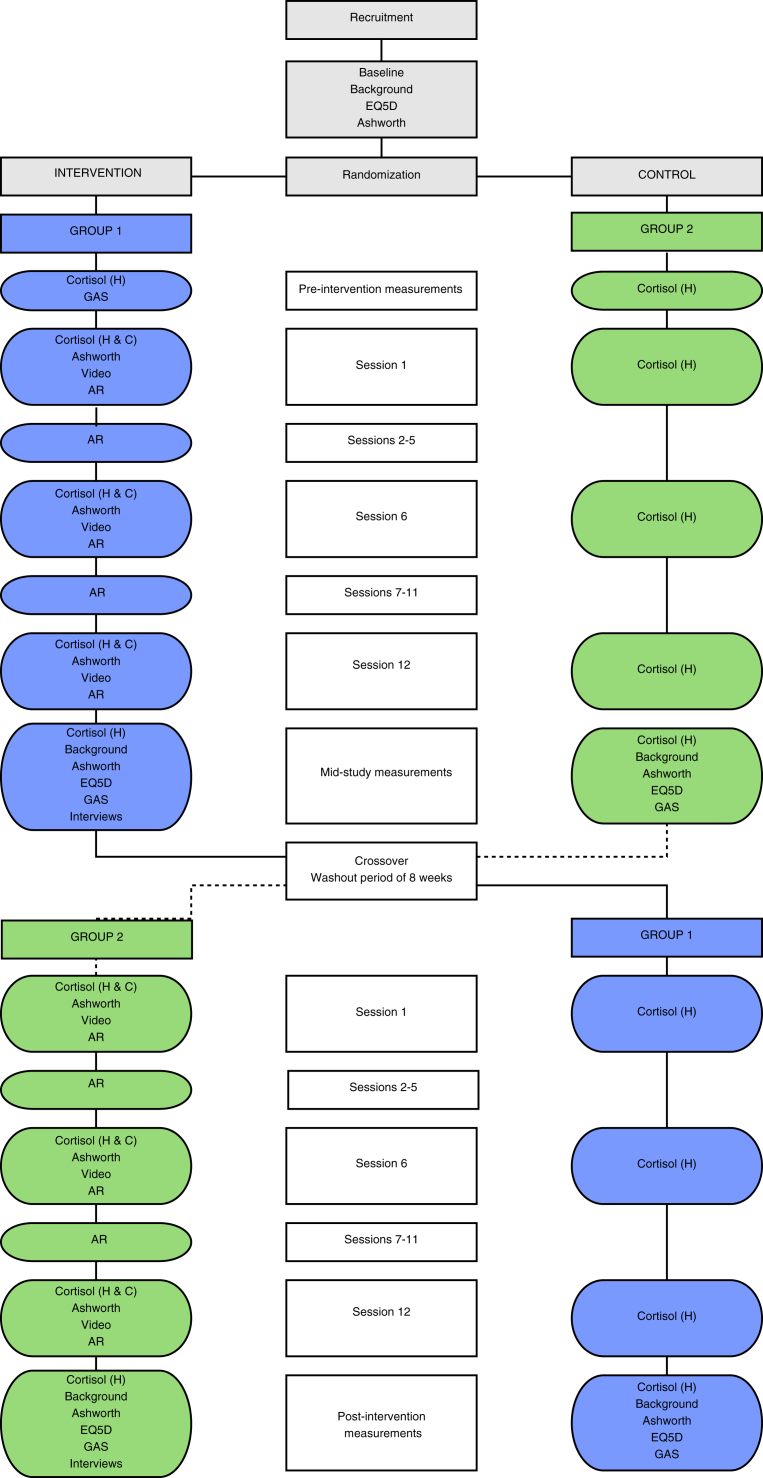
Study flow diagram of the evaluation of the Structured Water Dance Intervention (Background = Demography and physical health variables; Ashworth = Modified Ashworth Scale; EQ-5D = EuroQol five-dimension scale; AR = Assistants ratings; H = Home; C = Centre).

Since the prevalence of PIMD is very low, we will assess SWAN in a multicenter randomized intervention study enrolling people with PIMD at adult habilitation centers in four Swedish regions. To further increase the study's statistical power, we have applied a crossover design, as shown in Figure 1. At each center there will be two groups: an early intervention group (Group 1) and a late intervention group (Group 2). Group 1 will receive the intervention when they are entered into the study. Group 2 act as a control group and will be instructed to continue with their normal activities. Thereafter, Group 2 will receive the same intervention as Group 1, while Group 1 will be instructed to return to their normal activities. Thus, all participants will complete both control and intervention conditions.

### 2.2 Study sample

#### 2.2.1 Recruitment and eligibility of study participants

Selection and invitation of the participants will take place locally at each participating center. A licensed physiotherapist at the center selects potential participants based on the following inclusion criteria: (i) having a PIMD, i.e. having a profound intellectual disability (the level of intellectual functioning comparable with an estimated IQ of 20 or below, which in adults corresponds to a mental age below 3 years [14]), as well as having severe sensory and motor impairments corresponding to level IV–V in the Gross Motor Function Classification System [15], (ii) being an adult (18 years or older) and (iii) not finding discomfort with activities in water. Exclusion criteria are: (i) having severe hearing loss (hearing the music is a prerequisite for taking part in the intervention) and (ii) having infections or wounds that will be infectious in the pool. Due to their level of disability, all participants have a legal guardian; before participating in the study, the participant's legal guardian will give written consent after reading written information and being informed by the responsible researchers via telephone or in person.

#### 2.2.2 Sample size

Spasticity and cortisol are the primary outcomes of the study. As relevant data on spasticity and cortisol from previous research in the field is lacking, the power analysis is based on available spasticity data from the five persons who participated in the pilot study in 2017. Given that the estimated effect size and standard deviation of the participants in the pilot study are representative of the population, the sample size calculations shows that, with a power of 80% and an alpha of 5%, at least 40 participants are required in order to detect a clinically significant improvement (20%) in estimated spasticity after completed SWAN.

#### 2.2.3 Randomization

At each of the four centres, the participants are randomized into the early or late intervention group by minimization [16] based on gender, age and spasticity at baseline, using the MINIM software [17].

### 2.3 The SWAN program

#### 2.3.1 Multi-disciplinary research team

The research team for this project will consist of members from various disciplinary backgrounds including physiotherapy, psychology, sociology, rehabilitation, dance pedagogy, disability research, speech therapy, health economics, and project management. In addition, each participating center will have a local project administrator who is responsible for all practicalities regarding SWAN at the center.

#### 2.3.2 Program design

SWAN is performed as a group activity with 4–5 participants in a warm pool. The SWAN sessions are held once a week for 3 months (12 sessions in total). Each SWAN session takes 40 min and involves a playlist of nine songs. Each song is chosen to stimulate the rhythm of the movements performed, to support relaxation and to emphasize different emotions. Passive movement of different body parts is performed throughout the session. A support person accompanies each participant and functions as a dance partner. The SWAN sessions are led by two SWAN leaders (physiotherapists or assistant physiotherapists). One of them leads the group from the poolside and the other one is in the water to assist the participant and the participant's support person in order to optimize the experience of the activity. Participants use swim collars and other floating devices for an optimal floating position and for safety reasons. A more thorough description of the SWAN method can be found elsewhere [18].

During the study, a physiotherapist in the research team, who is experienced in leading SWAN groups, will be available by phone and e-mail and will also visit each participating center five times for monitoring, to perform measurements and to discuss issues or problems that arise. To ensure that SWAN is implemented in the same way in all participating centers, all SWAN leaders are trained in the theory, method and practice of SWAN. SWAN leaders also receive training in how the measurements are performed and how questionnaires are filled in. Prior to the intervention start, all participants visit the pool with their assistant to become familiar with the facilities and to ensure the working of the assistive devices and other practical support that the participant will need during the intervention.

#### 2.3.3 Health economics and implementation

Cost-effectiveness analyses will be carried out in order to give recommendations on how resources should be used to give the greatest health benefits in proportion to the costs of the intervention. Assuming that SWAN shows positive health economic effects, it is of great value to disseminate knowledge on SWAN to health care providers. Therefore, the project will study the implementation process in order to identify impeding and facilitating factors among the participating health care providers. SWAN will adhere to the RE-AIM framework [19] developed to identify systematically the factors that contribute to the implementation and survival of interventions.

### 2.4 Data collection

The project uses mixed methods, with both quantitative and qualitative data, to answer the study questions [12]. As shown in Table 1, the project uses a combination of overlapping quantitative measures to obtain reliable evaluations of the intervention effect in combination with interviews with the participants' assistants, SWAN leaders and center managers. The local project manager is responsible for monitoring and collecting the assistant's rating questionnaire after each SWAN session. However, cortisol measurements, video recordings and measurement of spasticity at the first, sixth and twelfth SWAN session as well as the administration of Goal Attainment Scaling (GAS) and interviews with staff and managers will be handled by the research team.

**Table 1:** Study variables.

Variable	Data collection methods		
	Direct measurement	Observation	Questionnaires/interviews
Stress	Salivary cortisol		Assistant's ratings
Spasticity	Physiotherapist's assessment		Assistant's ratings
Alertness		Video (facial expression, gaze, movement)	
Pain		Video (facial expression)	Assistant's ratings
Social interaction		Video (facial expression, gaze, movement)	Assistant's ratings
Well-being		Video (facial expression)	Assistant's ratings
Health-related quality of life			EQ-5D
Health economics	Salivary cortisol		EQ-5D and Assistant's ratings
Goal attainment			Goal Attainment Scaling
Experience of SWAN			Interviews with assistants and SWAN leaders
Implementation of SWAN			Interviews with managers

(SWAN = Structured Water Dance Intervention; EQ-5D = EuroQol five-dimension scale).

### 2.5 Measures

Demographic and medical data will be collected from the participant's medical records and through interviews with relatives and staff.

#### 2.5.1 Primary outcomes

The *Modified Ashworth Scale* [20] is a standardized procedure for measuring the resistance of an extremity in passive motion over a joint, rated on a six-point scale from 0 (no increase in muscle tone) to 5 (limb is rigid in flexion or extension). The scale has high inter-rater reliability [21] and concurrent validity [22], and is appropriate for measuring muscle tone in persons with PIMD [23]. All measurements are performed by the research team physiotherapist and each measurement takes 3–5 min to complete.

*Cortisol* level in saliva will be used as an index of stress [24]. There are no studies on diurnal cortisol of people with PIMD. However, studies on similar populations indicate that diurnal cortisol curves are within the normal range [25]. Thus, a given person tends to have a consistent diurnal rhythm but the cortisol levels may vary across individuals. Therefore, it is important to ensure individual reference points with different stress states [26]. Since cortisol levels tend to peak in the early morning hours and be at their lowest around midnight, measurements will be conducted in the morning when the participants are still in bed and in the evening at bedtime [27]. Cortisol will be measured on 16 occasions during the study (see Table 2), including morning and evening measurements performed at home one week prior to the first SWAN session and at home one week after the last SWAN session. These measurements represent the pre- and post-intervention measurements. During the intervention, cortisol will be measured in connection with the first, sixth and twelfth SWAN session. On these days, the measurements will be made four times [1]: at home in the morning [2], at the pool immediately before entering the pool [3], immediately before the participant leaves the pool after the SWAN session has ended and [4] at home in the evening. This procedure allows us to evaluate whether the SWAN intervention as a whole has an effect on the participants' stress levels (comparing differences from pre-intervention to post-intervention measurements between the intervention and control conditions) and whether the individual SWAN sessions have an effect on the participants’ stress level (comparing poolside measurements before and after the first, sixth and twelfth SWAN session).

**Table 2:** Cortisol saliva measurements.

	One week before	Session 1	Session 6	Session 12	One week after
Morning (on waking) at home	IC	IC	IC	IC	IC
Before entering the pool		I	I	I	
Before leaving the pool		I	I	I	
Evening (bedtime) at home	IC	IC	IC	IC	IC

I = intervention group, C = control group.

#### 2.5.2 Secondary outcomes

##### 2.5.2.1 Assistants’ rating questionnaire

This questionnaire was designed specifically for this study and consists of five statements regarding the participant's spasticity, pain, stress, social interaction and well-being. The assistant rates the participant before, during and after each SWAN session. Each statement is scored on a five-point Likert-type scale from ‘Strongly disagree’ to ‘Strongly agree’.

##### 2.5.2.2 Video recording

Each participant will be video-recorded in the pool on three occasions (on the first, sixth and twelfth SWAN session). The video recording will be made by the research team. From the video recordings, ratings will be made of each participant's expressions of pain, well-being and social interaction or contact, using a structured assessment protocol. Since the behaviors of the participants can be idiosyncratic, relatives and staff will assist in the interpretation of the video recordings. The video material will be assessed by two independent raters and the inter-rater reliability will be measured.

##### 2.5.2.3 Goal Attainment Scaling

(GAS) [28] measures the degree to which a person achieves identified goals with a treatment. The degree of fulfilment of the targeted goal is scored on a five-point scale from -2 (‘much worse than expected’) to +2 (‘much better than expected’), where 0 is the expected target [29]. In order to evaluate the study participant's individual goal achievement, at least one goal is established for each participant. Before the intervention, the goals of each participant are decided after discussions among the research team physiotherapist, the project coordinator and the person who best knows the participant (such as a member of staff or a relative). The assessment takes place after the sixth (mid-intervention) and the twelfth (last) SWAN session. GAS is a well-established and frequently used method in Sweden [30].

##### 2.5.2.4 EuroQol five-dimension scale

The *EuroQol five-dimension scale* (EQ-5D) [31] is a standardized instrument that measures quality of life in five dimensions (mobility, personal care, ordinary activities, pain/discomfort and anxiety/depression). Each dimension is scored on a five-point Likert scale, from 1 (‘no problems’) to 5 (‘unable to/extreme problems’). From the responses on the five dimensions, a preference-based index value that can vary between -1 and +1 is calculated. The value + 1 correspond to full health and 0 corresponds to death. The scale thus allows health conditions that are valued as worse than death. EQ-5D also contains a visual analogue scale of 100 mm, on which perceived current health is scored on a scale from 0 (‘worst possible health’) to 100 (‘best possible health’). The instrument has been validated in many countries as well as on several different disease states and disabilities [31]. It is generally considered good for capturing low quality of life [32], which makes it suitable for use on people with PIMD. EQ-5D was developed as a self-assessment instrument, but since self-assessment is not feasible in the present study, we will instead use the proxy version of EQ-5D [33], where the assessments are made by the person who best knows the participant (such as a member of staff or a close relative).

##### 2.5.2.5 Qualitative data

Focus groups with support persons and SWAN leaders will be conducted on completion of the intervention in order to gain insight into their experiences of the intervention. Each focus group will consist of five to seven people and is planned to last for approximately 1½ to 2 h. The focus group interviews will follow an interview guide and be audio recorded and transcribed verbatim. All focus groups will be facilitated by two members of the research team. In addition, semi-structured interviews will be conducted with managers of the centres in order to explore their experiences of opportunities and obstacles for the implementation of interventions in general and SWAN in particular. The inclusion of qualitative data will provide in-depth information on support persons', SWAN leaders' and mangers’ experiences; it will also give insights into the underlying processes that influence the feasibility of the intervention, such as what worked, what did not work, the perceived impact of SWAN and how to successfully implement SWAN in health care organizations.

### 2.6 Data management process

#### 2.6.1 Cortisol

The saliva collected in the home environment will be stored in a freezer (-18° Celsius) at the participants home and collected from all participants at the two follow-ups. The saliva tubes will be transported to the laboratory and stored according to prescribed procedures.

#### 2.6.2 Assistants’ ratings

The completed forms from each session will be stored by the local SWAN leaders and collected by the research team at the two follow-ups and stored in a locked safe at the principal investigator's workplace.

#### 2.6.3 GAS and EQ-5D ratings

The completed forms will be stored in a locked safe at the principal investigator's workplace.

#### 2.6.4 Audio-visual data

Video recordings of both groups from sessions 1, 6 and 12, as well as the audio recordings and the transcriptions of the interviews with staff and managers, will be stored on password-protected servers at the principal investigator's workplace.

### 2.7 Data analyses

Primary and secondary outcomes will be analyzed using multilevel linear mixed models [34], adjusting for the dependency of repeated measures and the hierarchical structure of the study as well as its adequacy in handling missing values. Time-invariant and time-varying covariates may also be included as predictors. Data will be analyzed according to intention to treat. However, the effects of the intervention on primary and secondary outcomes will also be evaluated regarding the number of sessions the participant attends. Qualitative data will be analyzed using inductive thematic analysis [35] for individual interviews and inductive content analysis for focus group interviews [36]. The results of the intervention will be reported according to the Template for Intervention Description and Replication (TIDieR) checklist and guide [37].

## 3 Discussion

This paper describes the SWAN study rationale and design, including details of the recruitment process, the intervention, the outcome measures, the health economics evaluation and the quantitative and qualitative data analyses. This is a multicenter study in four Swedish regions benefiting from a larger population of people with PIMD as potential participants and, consequently, a larger sample of staff and managers as informants for the qualitative interviews. If successful, the study will provide information on the effects and efficacy of SWAN for people with PIMD as well as information on cost effectiveness and on obstacles and enabling factors for the implementation of SWAN in health care settings. The strengths of the study include randomization by minimization to manage participant heterogeneity, the use of well-validated data collection methods, cost-effectiveness analyses and interviews with staff and managers to address opportunities and obstacles for the implementation of SWAN. Another strength is that the study responds to the need to address this specific target group and fill the research gap concerning customized interventions. However, a methodological limitation with the study is that the participants themselves, due to their profound disabilities, cannot give their personal, self-rated view on their participation in SWAN. Instead, we need to rely on other sources of information, such as objective measures, assessments and proxy ratings. In addition, although participants will be recruited through centers in four regions, thus covering a large population area, a possible limitation is that only people who can have two support persons accompanying them and resources to come to the centers will be included in the study. This may affect the generalizability of the results. Nonetheless, in conclusion it may be reasonable to assume that people with fewer resources will have similar needs and thus derive similar benefits from SWAN. Thus, if the objectives of the study are reached, it will provide a means to improve the health-related quality of life for people with PIMD, a group with substantial needs.

## Declarations

### Author contribution statement

L-O. Lundqvist, M. Matérne, A. Granberg, P. Arvidsson and A. Duberg: Conceived and designed the experiments; Analyzed and interpreted the data; Wrote the paper.

A. Frank: Conceived and designed the experiments; Performed the experiments; Analyzed and interpreted the data; Contributed reagents, materials, analysis tools or data; Wrote the paper.

### Funding statement

This work was supported by the Regional Research Council in the Uppsala–Örebro Region and the Research Committee of Region Örebro County.

### Competing interest statement

The authors declare no conflict of interest.

### Additional information

The clinical trial described in this paper was registered at ClinicalTrials.gov under the registration number NCT03908801.
